# Phenotypic stability and adaptability of wheat genotypes under organic and conventional farming systems over five years using AMMI and GGE biplot analysis

**DOI:** 10.3389/fpls.2025.1693316

**Published:** 2025-10-23

**Authors:** Nasser S. Al-Ghumaiz, Mohamad I. Motawei, Ahmed M. Aggag, Soleman M. Al-Otayk, Abdulmajeed A. Alzamil

**Affiliations:** ^1^ Department of Plant Production, College of Agriculture and Food, Qassim University, Buraydah, Qassim, Saudi Arabia; ^2^ Department of Environment and Natural Resources, College of Agriculture and Food, Qassim University, Buraydah, Saudi Arabia

**Keywords:** AMMI model, GGE biplot, genotype × environment interaction, wheat, stability, crop genetic diversity, organic and conventional fertilization

## Abstract

Organic agriculture is recognized for its sustainability, although it typically yields less than conventional systems. This study evaluated seven elite wheat genotypes (*Triticum aestivum* L.) over five years in a randomized complete block design with three replications, under both organic and conventional fertilization conditions. Integrated analyses using the AMMI model and GGE biplot revealed the significant effects of genotype, environment, and their interactions. The AMMI analysis showed that genotype IC8 achieved the highest mean yield (1.868 t ha^-1^) and the lowest AMMI stability value (ASV=0.474). This low ASV suggests high stability, indicating broad adaptability, especially under organic conditions. In contrast, Sids_12 (mean = 1.492 t ha^-1^; ASV=2.017) and LOCAL (mean = 1.304 t ha^-1^) exhibited great instability and specific adaptation. GGE biplot analysis explained 75.46% of the total variation (PC1 = 57.09%, PC2 = 18.37%), further confirming IC8’s stable performance across both systems while identifying P5 and IC17 as particularly responsive under conventional fertilization. These findings provide a basis for selecting wheat genotypes that balance high yield and stability, informing breeding strategies for sustainable crop production in both organic and conventional systems.

## Introduction

Organic agriculture is gaining popularity due to its sustainability and environmental benefits, such as improved soil health, lower chemical inputs, and increased biodiversity (Mäder et al., 2002; Reganold and Wachter, 2016). However, one of the most significant issues connected to organic farming is that it is generally less productive than conventional systems (Ponisio et al., 2015; Seufert et al., 2012). Wheat (*Triticum aestivum* L.) is a staple crop and the primary source of calories for a vast section of the world’s population. It is also critical to food security. As a result, increasing wheat yield while remaining sustainable, is crucial for fulfilling future food demands (FAO, 2020; Ritchie and Roser, 2022). Understanding genotype × environment (G×E) interactions is crucial for designing cultivars that thrive in various agricultural contexts (Kumar et al., 2021; Yan and Tinker, 2006). Recent improvements in statistical models, such as the additive main effects and multiplicative interaction (AMMI) model and the genotype and genotype-by-environment interaction (GGE) biplot analysis, have provided useful tools for understanding these complicated interactions. These methodologies allow breeders to not only discover high-yielding genotypes, but also to assess their stability under changing environmental conditions (Singh et al., 2019; Yan et al., 2007). Organic and inorganic fertilization approaches can influence Se and Zn concentrations, potentially affecting crop nutrition and human health (Alloway, 2008; Lyons et al., 2017). Abd-Elmoniem et al. (2025); Al-Ghumaiz et al. (2020) evaluated the impacts of fertilization methods on wheat mineral composition, focusing on the significance of fertilization strategies in increasing nutrient density.

Few studies have comprehensively evaluated the long-term stability of wheat (*Triticum aestivum* L.) genotypes under various fertilization regimes. Research assessing genotype performance across multiple years, particularly under conventional and organic fertilization systems, remains limited. Such studies are essential for stable, high-yielding cultivars capable of adapting to diverse agronomic conditions. The objectives of this study were to (i) evaluate the patterns and magnitude of G×E interactions affecting yield stability of seven elite wheat genotypes across five successive growing seasons (2019-2023) under conventional and organic fertilization systems, and (ii) identify wheat genotypes that combine highly productivity with stability across years and fertilization methods to support breeding programs for sustainable wheat production in arid and semi-arid environments.

## Materials and methods

### Experimental site

Field experiments were conducted over five consecutive growing seasons from 2019 to 2023 at the Agricultural Research and Experimental Station of Qassim University, Buraydah, Saudi Arabia (26°18′28″ N, 43°46′ E). The site is characterized by sand texture with low salinity (electrical conductivity, EC=1.5 dSm^-1^), low organic matter content (0.4%), and an alkaline pH of 8.1. The irrigation water used throughout the study had an EC of 1.7 dSm^-1^ and a pH of 7.8.

### Experimental design and treatments

The study evaluated the performance of seven wheat (*Triticum aestivum* L.) genotypes (**Table 1**) under two fertilization regimes: organic (O) and inorganic (N). Thus, experimental factors were:

**Table 1:** Seven wheat genotypes used in this study.

Genotype name	Source
Yocora Rojo (YR)^†^	USA
LOCAL^‡^	KSA
P3 (AUS-030851)	Australia
P5 (AUS-030852)	Australia
IC8 (Line-2-ICARDA-1^st^ RDRN0607)	ICARDA
IC17 (Line-56 ICARDA-1^st^ RDRN0607)	ICARDA
Sids_12	Egypt

^†^Yocora Rojo (YR): commercial genotype commonly cultivated in Saudi Arabia.

^‡^Local genotype (Sama).

- Factor 1 (Genotypes): Seven wheat (*Triticum aestivum* L.) genotypes.
- Factor 2 (Fertilization): Two regimes-organic (O) and inorganic (N).

Trials were laid out in a randomized complete block design (RCBD) with three blocks. Each block contained 14 plots (7 genotypes × 2 fertilization regimes). Each plot measured 3 m² (1.5m × 2.0m) and comprised 10 rows spaced 25cm apart. Wheat seeds were sown at a seeding rate of 45kg ha^-1^.

The planting dates for the five seasons were as follows: December 2019, November 30, 2020, December 2021, December 2022, and December 2023.

### Fertilizer application

Fertilizer application followed soil test recommendations. In the inorganic treatment, urea, diammonium phosphate (DAP), and potassium sulfate were applied at rates of 124kg N ha^-1^, 92kg P_2_O_5_ ha^-1^, and 57kg K_2_O ha^-1^, respectively. For the organic treatment, well-decomposed cow manure was applied at a rate of 10t ha^-1^ one month before sowing. The organic amendment was analyzed according to the methodology of (Sparks et al., 2020), revealing an N:P:K ratio of 0.5:0.21:0.5.

### Environmental and soil conditions

**Figure 1:**
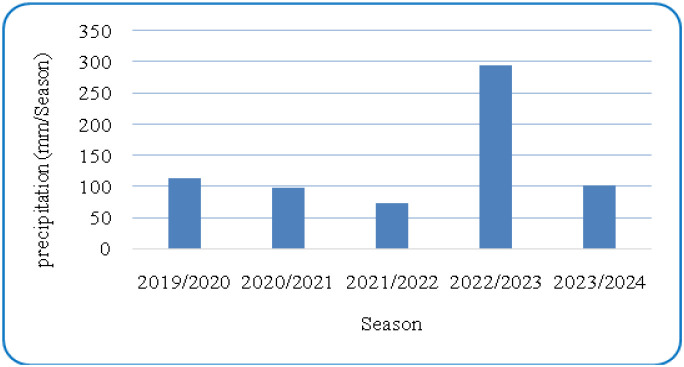
Five-years average (2019–2023) of annual precipitation in Qassim region.

**Figure 2:**
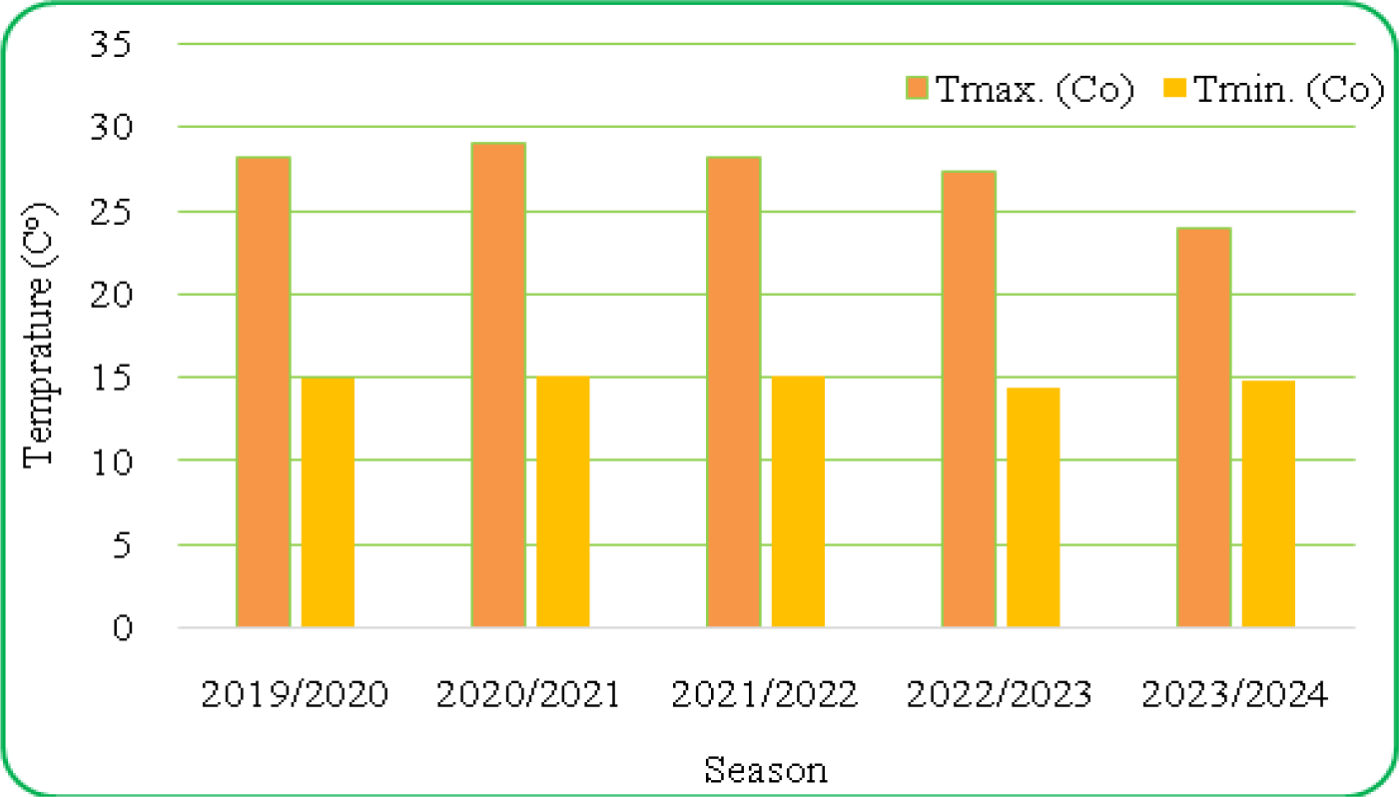
Average of the maximum and minimum temperature from 2019 to 2023 in the experimental field consistently across environments.

**Figures 1** and **2** present the meteorological data for the study period (2019–2023), including maximum and minimum temperatures and total rainfall. A summary of environmental conditions across different years is provided in **Table 2**. Soil physical and chemical properties of the experimental site are detailed in **Table 3**.

**Table 2:** Environmental conditions* across different years and fertilizations (organic (O) and inorganic (N) based on precipitation and temperature variables.

Number	Environment	Precipitation (mm)	Tmax (°C)	Tmin (°C)
1	2019-N	146.5	26.64	14.01
2	2019-O	146.5	26.64	14.01
3	2020-N	113.5	28.19	14.84
4	2020-O	113.5	28.19	14.84
5	2021-N	96.6	28.16	15.07
6	2021-O	96.6	28.16	15.07
7	2022-N	72.5	28.19	14.84
8	2022-O	72.5	28.19	14.84
9	2023-N	294.8	27.34	14.34
10	2023-O	294.8	27.34	14.34

*TerraClimate dataset, Climatology Lab, available at: http://www.climatologylab.org/terraclimate.html.

**Table 3:** Soil chemical and physical analyses of the two experimental sites.

Site	Chemical analysis						Particle size distribution (%)		
	K (ppm)	P (ppm)	N (ppm)	OM**	pH	EC (dS/m)	Clay	Silt	Sand
Conventional site*	34	33.1	15.7	0.4	8.1	1.3	0.9	4.2	94.9
Organic site*	36.5	22.1	52.5	0.4	7.9	1.7	1.0	4.5	94.5

*Qassim University Agricultural Research Station.

**OM= organic matter.

### Statistical analysis

The AMMI model (Gauch, 1992, 2013) was used to calculate the yield stability of the elite spring wheat genotypes under organic and inorganic fertilizations. Principal component analysis (PCA) was used to determine the multiplicative effects of GEI after the AMMI model first fitted the additive effects for the primary influences of the genotypes (G) and environment (E). Biplot graphs were used to display the AMMI findings. According to (Nowosad et al., 2016), the AMMI model can be stated using the formula below:


$$Y_{ge}=\mu +\alpha_{g}+\beta_{e}+\sum_{n=1}^{N}\lambda_{n}\gamma_{gn}\delta_{en}+Q_{ge}$$

where N is the number of PCA axes kept in the adjusted model; λ_n_ is the eigen value of the PCA axis, n; γ_gn_ is the genotype score for the PCA axis, n; δ_en_ is the score eigenvector for the PCA axis, n; y_ge_ is the trait mean of a genotype g in environment e; μ is the grand mean; α_g_ is the mean genotype deviation; β_e_ is the mean environment deviation; and Q_ge_ is the residual, including the AMMI noise and pooled experimental error.

AMMI analysis was carried out using the “AMMI” function from the GenStat statistical software, version 19 (GenStat, 2019). The AMMI stability value (ASV) coefficient (Purchase et al., 2000) was used to evaluate the genotype stability. The more stable the genotype in the conditions under study, the lower the ASV. Each genotype was assigned a genotype selection index (GSI), which is the total of the ASV and yield stability index (YSI) ranking positions (Farshadfar, 2008).

A GGE biplot was constructed using principal component analysis, wherein the scores of genotypes were multiplied by the corresponding environmental scores to produce a two-dimensional representation (Yan and Wu, 2008). This approach enabled a simultaneous assessment of both the main effects of genotypes and their interactions with the environment.

## Results

### Genotype stability based on AMMI analysis

The ANOVA results (**Table 4**) obtained for the AMMI model clearly indicate the significant effects of both genotype and environment on the traits under consideration, supporting the hypothesis that performance varies across different conditions and genetic backgrounds. The interaction between genotypes and environments was also significant, with the first principal component (IPCA1) showing a high variance ratio (v.r. = 4.74; *p<* 0.001). The second principal component (IPCA2) was also significant (v.r. = 2.14; *p*<0.0473), indicating that specific genotype responses varied across environments.

**Table 4:** ANOVA for AMMI model.

Source of Variance	d.f.	Sum of squares	Mean squares	F	*p-*value
Genotypes	6	2.146	0.3577	3.13	0.0105
Environments	9	14.775	1.6416	14.36	<0.001
Interactions	54	6.175	0.1144		
IPCA 1	14	3.413	0.2438	4.74	<0.001
IPCA 2	12	1.323	0.1103	2.14	0.0473
Residuals	28	1.440	0.0514		

d.f., degrees of freedom; IPCA, principal component of interaction.

### Genotype means and scores

The mean performance and IPCA scores for each genotype revealed differences in adaptability and stability (**Table 5**). IC8 (mean = 1.868t ha^-1^) exhibited the highest mean performance among the tested genotypes and had a positive IPCA score in the first principal component, indicating favorable interactions with certain environments. IC17 (mean = 1.758t ha^-1^) also performed well, with a positive IPCAg1 score, suggesting its relative stability across environments. In contrast, LOCAL (mean = 1.304t ha^-1^) had the lowest mean performance and negative IPCA scores in both components, indicating its performance was less favorable across environments. Sids_12 had a mean of 1.492t ha^-1^, with a high positive IPCAg2 score, suggesting that it performed well under certain environmental conditions but may not be stable across all environments.

**Table 5:** Mean performance and principal component scores (IPCAg1 and IPCAg2) of wheat genotypes across all environments.

Genotype	Number	Mean (t ha^-1^)	IPCAg1	IPCAg2
IC17	1	1.758	0.28815	0.05984
IC8	2	1.868	0.09287	-0.40885
LOCAL	3	1.304	-0.63680	-0.19282
P3	4	1.539	-0.19608	-0.24409
P5	5	1.728	0.51962	-0.40490
Sids_12	6	1.492	-0.75479	0.52550
YR	7	1.610	0.68704	0.66533

### AMMI stability values

The ASVs provided insights into the performance and adaptability of genotypes across different environments (**Table 6**). Lower ASVs indicated greater stability, which means that the genotype is less affected by environmental changes. The AMMI analysis identified IC8 (ASV=0.474) as the most stable genotype with the highest mean yield (1.868t ha^-1^), indicating consistent performance across varying conditions. In contrast, Sids_12 (ASV=2.017) was ranked the least stable, suggesting it is more sensitive to environmental changes despite a mean yield of 1.492t ha^-1^. P3 and IC17 ranked second and third in stability, also showing relatively high mean yields of 1.539 and 1.758t ha^-1^, respectively. This balance between stability and yield performance is valuable for selecting genotypes for breeding programs. Conversely, LOCAL (ASV=1.654) and YR (ASV=1.893) exhibited high instability, indicating that they may perform well only under specific conditions but lack broad adaptability.

**Table 6:** AMMI stability values, rankings, and mean performance of wheat genotypes.

Rank	Genotype	Number	Stability (ASV)	Mean (t ha^-1^)
1	IC8	2	0.474	1.868
2	P3	4	0.562	1.539
3	IC17	1	0.746	1.758
4	P5	5	1.400	1.728
5	LOCAL	3	1.654	1.304
6	YR	7	1.893	1.610
7	Sids_12	6	2.017	1.492

### Identification of promising genotypes across environments

The AMMI analysis revealed the first four genotype selections for each environment based on their performance (**Table 7**). The highest mean yield was recorded in environment 2023-N (2.208t ha^-1^), followed closely by 2020-N (2.204t ha^-1^) and 2019-N (2.195t ha^-1^). These environments demonstrated favorable conditions for high-yielding genotypes. Conversely, environments such as 2022-O (0.908t ha^-1^) and 2021-O (0.945t ha^-1^) had the lowest mean performance, indicating suboptimal conditions for yield expression. Genotype IC8 emerged as the most frequently selected across environments, appearing in the top four rankings in nine out of ten environments. This highlights its broad adaptability and stability across varying conditions. Other frequently selected genotypes include IC17, P5, and YR, indicating their suitability for specific environments. Sids_12 was also prominent in environments such as 2022-O, 2020-N, and 2019-N, suggesting its potential for high performance in targeted conditions. The AMMI interaction scores varied significantly across environments. Environments such as 2023-O (0.8505) and 2023-N (0.6207) had high scores, demonstrating strong genotype-by-environment interaction effects. Lower interaction scores in environments such as 2022-N (0.0048) and 2021-O (-0.2990) suggest reduced genotype-by-environment interaction, emphasizing the role of stable environmental conditions.

**Table 7:** Top four AMMI selections for each environment based on mean performance and scores.

Number	Environment	Mean	Score	1	2	3	4
10	2023-O	1.534	0.8505	YR	P5	IC17	IC8
9	2023-N	2.208	0.6207	P5	IC8	IC17	YR
5	2021-N	1.460	0.2525	IC8	P5	IC17	YR
2	2019-O	1.658	0.0800	YR	IC17	IC8	P5
7	2022-N	1.321	0.0048	IC8	IC17	YR	P5
6	2021-O	0.945	-0.2990	IC8	Sids_12	IC17	P3
8	2022-O	0.908	-0.3245	Sids_12	IC8	IC17	P3
4	2020-O	1.707	-0.3366	IC8	P5	P3	LOCAL
1	2019-N	2.195	-0.4084	Sids_12	IC8	IC17	P3
3	2020-N	2.204	-0.4401	Sids_12	IC8	IC17	P3

### Genotype stability based on GGE biplot analysis

**Figure 3:**
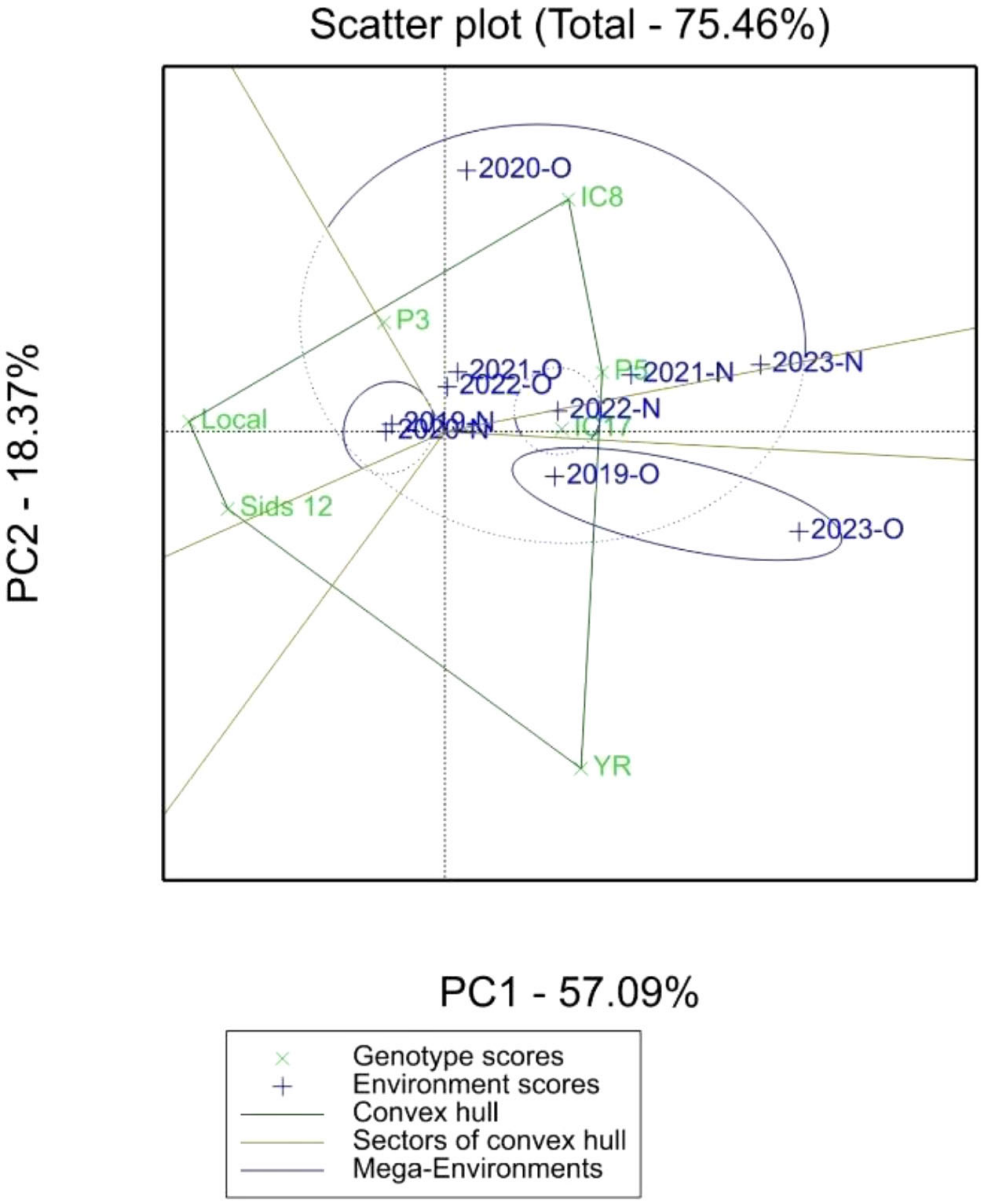
Polygon view of GGE biplot (which-won-where model) showing 7 elite wheat genotypes in organic (O) and conventional fertilization (N) environments.

The polygon view of the GGE biplot (**Figure 3**) provides a comprehensive visual representation of the “which-won-where” model, enabling the identification of wheat genotypes that perform optimally under specific environments (organic and conventional fertilization systems). The biplot presented in **Figure 3** summarizes the principal component analysis (PCA) of the genotypic and environmental data, explaining 75.46% of the variation, with PC1 and PC2 contributing 57.09% and 18.37%, respectively. The environments are grouped into two distinct mega-environments based on their proximity to vertex genotypes: Mega-Environment 1 (Conventional Environments) includes environments 2023-N, 2021-N, 2022-N, and 2019-N. Genotypes located at the vertices of the polygon, such as P5 (AUS-030852) and IC17 (Line-56 ICARDA-1st RDRN0607), demonstrated superior adaptability and yield performance in their respective environments. These genotypes are likely the most suited to the environmental conditions and nutrient availability provided by the specific fertilization system. Mega-Environment 2 (Organic Environments: 2020-O, 2019-O, and 2023-O) was dominated by genotype IC8. Genotypes IC8, IC17, and P5 were the primary vertex genotypes, suggesting superior performance in their respective mega-environments. Other genotypes, such as YR, Sids_12, and LOCAL, were positioned further away from the polygon edges, indicating either lower interaction or suboptimal performance in specific environments. Genotypes close to the biplot origin (e.g., P3) demonstrated greater stability.

### Mean vs. stability

**Figure 4:**
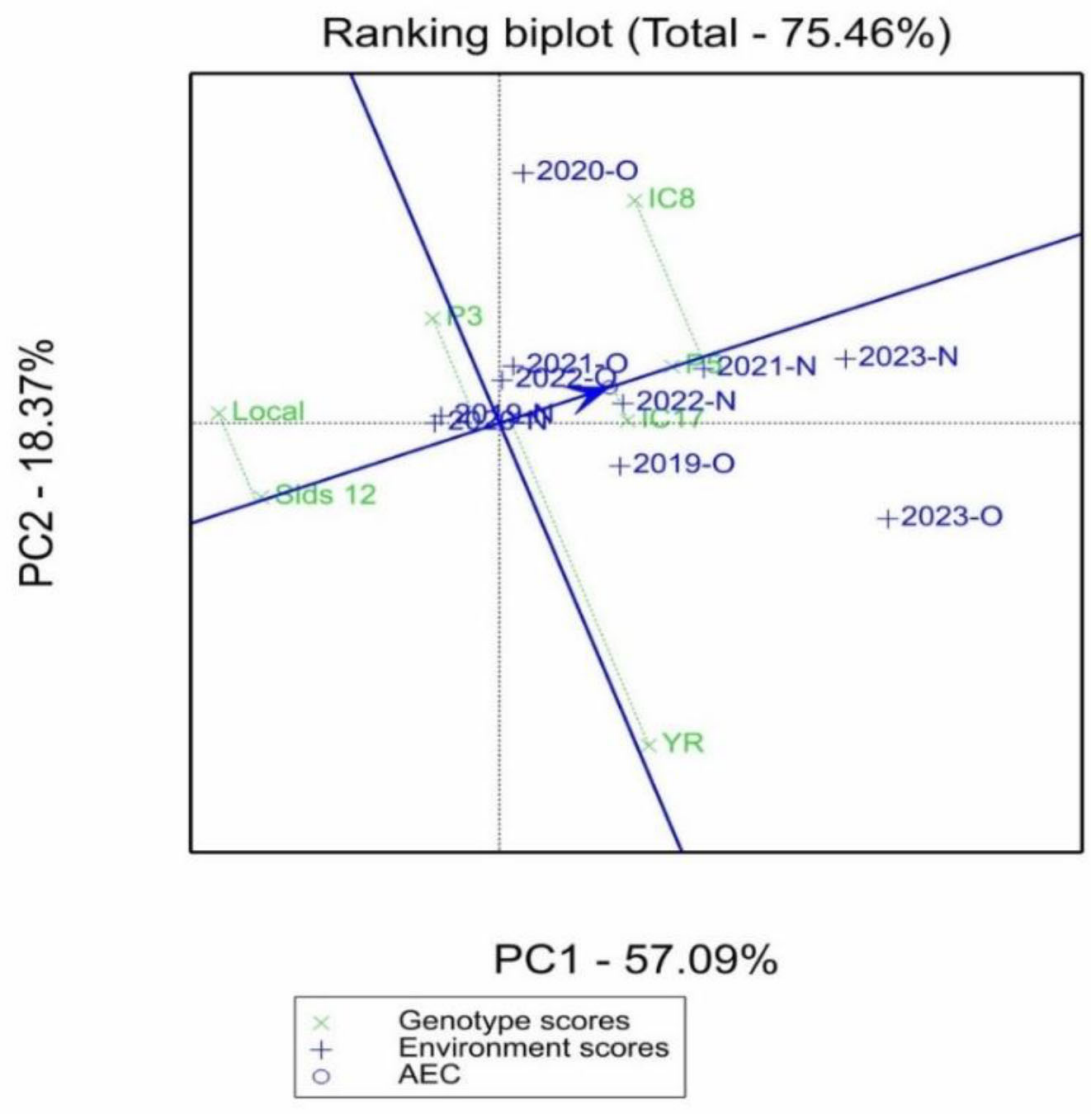
Mean vs. stability view of GGE biplot showing the mean performance and stability of seven elite wheat genotypes in organic (O) and conventional fertilization (N) environments.

**Figure 4** displays a ranking biplot that explains 75.46% of the total variation in genotype and environmental scores, with the first principal component (PC1) explaining 57.09% of the variance and the second component (PC2) explaining 18.37%. Genotypes are represented by green crosses, while environments are depicted by blue crosses with vectors. The average environment coordination (AEC) axis, shown as a blue line, indicates the average performance of genotypes across environments. The AEC circle helps visualize the distance of environments from the average performance. The genotypes “IC8,” “LOCAL,” and “P3” are spread close to the AEC, indicating stability in their performance across different environments. Conversely, “YR” and “IC8” are separated along PC1 and PC2, suggesting more specific adaptation to certain environments. Environments such as “2020-O” and “2023-O” are farther along the AEC axis, suggesting that they either posed challenges or provided advantages that influenced genotype performance.

## Discussion

This study showed that the performance of wheat was influenced by both genotypic variances and environmental variables, with a strong G×E interaction. This finding is consistent with earlier research demonstrating that diversified genotype selection can increase total crop output (Zobel et al., 1988). The significant influence of environmental factors underscores the idea that genotype performance is extremely context-dependent, which is important in modern plant breeding practices (Kumar et al., 2021; Yan and Tinker, 2006). The significant interaction (F=4.74; p< 0.001) highlighted the need to consider both genetic and environmental factors when planning breeding programs to achieve consistent and high-yielding genotypes.

The AMMI model showed that the first two interaction principal component axes (IPCAs) account for the majority of variation in the G×E interactions. These findings support previous research showing the efficacy of the AMMI model in interpreting complicated relationships in multi-environment trials (Gauch, 1992, 2013). In our investigation, genotypes with low ASVs, such as IC8, P3, and IC17, produced high yields and performed consistently across conditions. In contrast, genotypes such as YR and Sids_12 had high ASVs and showed significant variability, indicating a preference for specific environmental circumstances over broad adaptability.

The ranking biplot contributed to our understanding by explaining 75.46% of the overall variation, with PC1 and PC2 accounting for 57.09% and 18.37%, respectively. The average environment coordination (AEC) axis represents average performance across situations. Genotypes along the AEC axis, such as IC8, LOCAL, and P3, indicated stability, but genotypes dispersed over both major components, such as YR, suggested particular adaptation to certain environmental conditions. The environmental conditions presented in **Table 2** revealed considerable variation between years, particularly in terms of precipitation which ranged from 72.5mm in 2022 to a 294.8mm in 2023. These variations, in combination with the use of organic (O) and inorganic (N) fertilizers, resulted in various environments that influenced genotype performance. Interestingly, environments “2020-O” and “2023-O” were located further down the AEC axis, suggesting that they presented difficulties or benefits that had a large impact on genotype performance. This trend aligns with research showing how important environmental influences are in influencing genetic responses (Yan et al., 2007). Particularly in low-input systems where reliable performance is crucial, genotypes like IC8, P3, and IC17 that consistently performed well in a variety of environments are interesting candidates for widespread cultivation (Baresel et al., 2008; Singh et al., 2019). However, highly variable genotypes, such as YR and Sids_12, might be targeted for certain management techniques or settings where their distinct performance profiles can be best utilized (Annicchiarico, 2002). The clustering of organic environments around genotypes such as IC8 suggests enhanced nutrient-use efficiency and stress tolerance, traits that are highly desirable for sustainable organic agriculture (Baresel et al., 2008). Al-Ghumaiz et al. (2020) showed that the ICARDA genotypes (IC8 and IC17) had the greatest Se and Zn concentrations, respectively, under organic fertilization.

When yield performance is integrated with stability measurements, it becomes clear that selecting genotypes simply based on yield might overlook important performance consistency characteristics. Vassileva et al. (2023) reported that cultivating drought-resistant wheat genotypes and understanding stability determinants could markedly contribute to enhancing wheat production and ensuring stable yields under low-input and stress-pone environmental conditions. ASV index, a measure of yield stability that takes into account genotype-by-environment interactions (Purchase et al., 2000) was especially valuable in this investigation. IC8 was the most promising contender due to its high mean yield and low ASV, suggesting strong performance throughout a wide variety of environmental conditions. This is critical for creating genotypes that can maintain productivity in various environments (Ahakpaz et al., 2021). In contrast, despite being genetically distinct, IC17 showed a balance between stability and yield. Its moderate yield, coupled with consistent performance across environments, suggests that it may offer specific adaptive benefits that are not captured by yield alone. Conversely, genotypes such as YR and Sids_12 may require targeted management practices or further breeding interventions to enhance their performance, as they either did not yield as highly or exhibited greater variability. These findings highlight the significance of using a dual-criteria selection approach that incorporates both yield potential and stability. Such an integrated strategy ensures that selected genotypes are not only high-yielding but also resilient under diverse environmental conditions, a critical requirement for sustainable cultivation and breeding programs (Crossa et al., 2017).

## Conclusion

The integration of AMMI analysis and GGE biplot visualization in this study provided a robust framework for evaluating the performance of genotypes under varying environmental conditions. Our results indicated that both yield and stability, as measured by ASV and mean yield, are crucial parameters in selecting genotypes for cultivation. Genotype IC8 emerged as the most promising candidate, combining high yield with stability, thereby offering broad adaptation to diverse environments. Similarly, IC17 also demonstrated a favorable balance between yield and stability, making it suitable for general cultivation. In contrast, genotypes such as YR and Sids_12, which displayed high variability, may be better suited for targeted environments where specific management practices can mitigate their instability. Future research should focus on integrating additional agronomic traits and exploring the molecular mechanisms underlying these interactions to further refine selection strategies for sustainable crop production.

## Data Availability

The original contributions presented in the study are included in the article/supplementary material. Further inquiries can be directed to the corresponding author/s.
